# Probable primary bloodstream infection caused by multidrug-resistant *Achromobacter piechaudii* in an apparently immunocompetent child: a case report

**DOI:** 10.1186/s12887-026-07498-8

**Published:** 2026-08-10

**Authors:** Yunfeng Xiang, Ling Duan, Daoxue Xiong, Xiangjun Liu

**Affiliations:** Department of Pediatric Critical Care, Chongqing University Three Gorges Hospital, Wanzhou, Chongqing, China

**Keywords:** Child, Achromobacter piechaudii, Bloodstream infection, Apparently immunocompetent, Multidrug-resistant, Case report

## Abstract

**Background:**

*Achromobacter piechaudii* is an extremely rare opportunistic pathogen in humans. Previous reports of clinical infection have mostly involved adults with underlying conditions such as malignancies or immunocompromised states.This report describes the probable first case of primary, multidrug-resistant *Achromobacter piechaudii* bloodstream infection in an apparently immunocompetent child.

**Case presentation:**

A previously healthy 10-year-10-month-old boy with no known immunodeficiency or chronic disease was admitted with intermittent headache and fever for 4 days, accompanied by chills, vomiting, and lethargy.He had no recent travel, sick contacts, or known exposure to contaminated water. Complete blood count showed elevated white blood cell count and neutrophil percentage. C-reactive protein and procalcitonin were markedly elevated. Empirical treatment with ceftriaxone was started on admission. On day 2 of hospitalization, blood culture indicated growth of Gram-negative bacilli. On day 3, the isolate was identified as *Achromobacter piechaudii* by MALDI-TOF MS (score > 2.3). Cerebrospinal fluid examination was normal, confirming primary bloodstream infection. Antimicrobial susceptibility testing showed a multidrug-resistant phenotype. The antibiotic regimen was switched to piperacillin-tazobactam after resistance to ceftriaxone was confirmed.The patient’s fever resolved, clinical symptoms disappeared, and the outcome was favorable.

**Conclusion:**

To our knowledge, this is the probable first case report of primary multidrug-resistant *Achromobacter piechaudii* bloodstream infection in an apparently immunocompetent child.This case suggests that clinicians should consider rare *Achromobacter* species when managing pediatric bloodstream infection without obvious immunodeficiency. The multidrug-resistant phenotype highlights the need for accurate species identification and susceptibility-guided therapy.

**Supplementary Information:**

The online version contains supplementary material available at https://doi.org/10.1186/s12887-026-07498-8.

## Introduction

*Achromobacter piechaudii* is an extremely rare pathogenic species. Clinical infections have been reported almost exclusively in adults with serious underlying diseases or immunocompromised conditions, and no invasive infection has been reported in apparently immunocompetent children. we searched PubMed, Scopus, Web of Science, and Google Scholar up to April 2026 using the terms “*Achromobacter piechaudii*”, “*Alcaligenes piechaudii*”, “bloodstream infection”, “bacteremia”, “child”, “pediatric”, and “immunocompetent”. No previous pediatric case was found.

Previously reported *Achromobacter piechaudii* strains were mostly susceptible to common antibiotics [1, 2]. Here we report the probable first case of primary bloodstream infection caused by multidrug-resistant *Achromobacter piechaudii* in an apparently immunocompetent child. This case extends the host range of invasive *A. piechaudii* infection to healthy children and alerts clinicians to the possibility of multidrug-resistant phenotypes in this rare pathogen.

## Case presentation

A 10-year-10-month-old boy was admitted with intermittent headache and fever for 4 days, accompanied by vomiting and chills. He had been previously healthy, with no history of recurrent infections, known immunodeficiency, or long-term use of immunosuppressive agents. He had no history of travel, swimming, trauma, injections, dental procedures, or exposure to contaminated water sources, animals, or soil in the preceding month. On admission, physical examination showed a temperature of 39 °C, heart rate 129 beats/min, respiratory rate 35 breaths/min, blood pressure 115/68 mmHg, and weight 26.6 kg. He was conscious but lethargic with poor response. Pharyngeal congestion was present. Cardiopulmonary and abdominal examinations were unremarkable. Nuchal rigidity was positive, while Kernig’s sign, Brudzinski’s sign, and pathological reflexes were negative. Laboratory tests: white blood cell count 11.62 × 10⁹/L, neutrophil percentage 95.3%, C-reactive protein 59.44 mg/L, procalcitonin 14.448 ng/mL. Chest X-ray and abdominal ultrasound showed no abnormalities.

The diagnoses considered on admission included fever of unknown origin, possible sepsis, and possible intracranial infection. Empirical anti-infective therapy with ceftriaxone sodium 2.0 g intravenously once daily was initiated. Lumbar puncture was performed. Cerebrospinal fluid (CSF) appeared clear, and opening pressure was normal. CSF white blood cell count was 3 × 10⁶/L, protein 0.11 g/L, glucose 6.0 mmol/L, and chloride 122.6 mmol/L.

On day 2 of hospitalization, the child’s nuchal rigidity resolved. Blood culture returned positive for Gram-negative bacilli. One set of blood cultures was drawn, consisting of two aerobic bottles; both bottles signaled positive after 14.2 h of incubation. On day 3, the blood culture isolate was identified using a matrix-assisted laser desorption ionization time-of-flight mass spectrometry (MALDI-TOF MS) system (Bruker MALDI Biotyper^®^) with the MBT IVD Reference Library (version 2023). The isolate was identified as Achromobacter piechaudii (score > 2.3). The database contains reference spectra for *Achromobacter piechaudii.* However, molecular confirmation by *nrdA* sequencing was not performed, which is a limitation.In vitro antimicrobial susceptibility testing was performed using the VITEK 2 system (AST-N335 card). Because the Clinical and Laboratory Standards Institute (CLSI) has not established species-specific breakpoints for Achromobacter, the results were interpreted using CLSI breakpoints for “Other Non-Enterobacterales”.For key antimicrobials, the minimum inhibitory concentrations (MICs) were further verified manually using the E-test method. The susceptibility results showed a marked multidrug-resistant phenotype: the isolate was susceptible to piperacillin and piperacillin-tazobactam, intermediate to minocycline, and resistant to amikacin, ceftazidime, ceftriaxone, cefepime, meropenem, aztreonam, levofloxacin, and trimethoprim-sulfamethoxazole. Imipenem MIC was 16 µg/mL, but no CLSI interpretive criteria exist for this organism, so only the MIC is reported. The complete MIC data are shown in Table 1. The isolate met the provisional criteria for multidrug resistance (resistance to at least one agent in three or more antimicrobial classes). A definite diagnosis of *Achromobacter piechaudii* bloodstream infection was made. The microbiological details are summarized in Table 2.Based on the susceptibility results, ceftriaxone was discontinued and replaced with the beta-lactamase inhibitor combination piperacillin-tazobactam (8:1 formulation) 3.0 g (piperacillin component) intravenously every 8 h (approximately 112.5 mg/kg/dose of piperacillin), infused over 30 min.The total duration of piperacillin-tazobactam therapy was 12 days.

**Table 1:** Antimicrobial susceptibility profile of the Achromobacter piechaudii isolate

Antimicrobial agent	MIC (µg/mL)	Testing method	Breakpoint used	Interpretation
Amikacin	64	VITEK 2 / E-test	Other Non-Enterobacterales (CLSI)	R
Ceftazidime	64	VITEK 2	Other Non-Enterobacterales (CLSI)	R
Ceftriaxone	64	VITEK 2	Other Non-Enterobacterales (CLSI)	R
Cefepime	64	VITEK 2	Other Non-Enterobacterales (CLSI)	R
Imipenem	16	VITEK 2 / E-test	No CLSI breakpoint	MIC only
Meropenem	16	VITEK 2 / E-test	Other Non-Enterobacterales (CLSI)	R
Aztreonam	64	VITEK 2	Other Non-Enterobacterales (CLSI)	R
Levofloxacin	8	VITEK 2 / E-test	Other Non-Enterobacterales (CLSI)	R
Trimethoprim-sulfamethoxazole	320	VITEK 2 / E-test	Other Non-Enterobacterales (CLSI)	R
Minocycline	8	VITEK 2	Other Non-Enterobacterales (CLSI)	I
Piperacillin	≤ 16	VITEK 2	Other Non-Enterobacterales (CLSI)	S
Piperacillin-tazobactam	≤ 16	VITEK 2 / E-test	Other Non-Enterobacterales (CLSI)	S

*MIC* Minimum inhibitory concentration, *CLSI* Clinical and Laboratory Standards Institute, *R* Resistant, *I* Intermediate, *S* Susceptible

Because species-specific breakpoints for A. piechaudii have not been established by CLSI, the breakpoints for “Other Non-Enterobacterales” (CLSI M100) were provisionally applied. No CLSI breakpoint for imipenem is available in this category; therefore, only the MIC value is reported. MIC values for key agents (carbapenems, piperacillin-tazobactam, trimethoprim-sulfamethoxazole, and fluoroquinolones) were confirmed by E-test

**Table 2:** Microbiological details of the bloodstream infection episode

Item	Detail
Blood culture sets drawn	1 set (2 aerobic bottles)
Positive bottles / total bottles	2 / 2 (both aerobic)
Time to positivity (hours)	14.2
Gram stain result	Gram-negative bacilli
Identification method	MALDI-TOF MS (Bruker Biotyper), score > 2.3
Repeat blood culture before antibiotic switch	No
Follow-up blood culture after antibiotic switch	Collected on day 8; finalized negative on day 13
Final clinical diagnosis	Primary bloodstream infection

*MALDI-TOF MS* Matrix-assisted laser desorption ionization time-of-flight mass spectrometry. *A. piechaudii* was identified with a score > 2.3 using the MBT IVD Reference Library (version 2023)

**Fig. 1:**
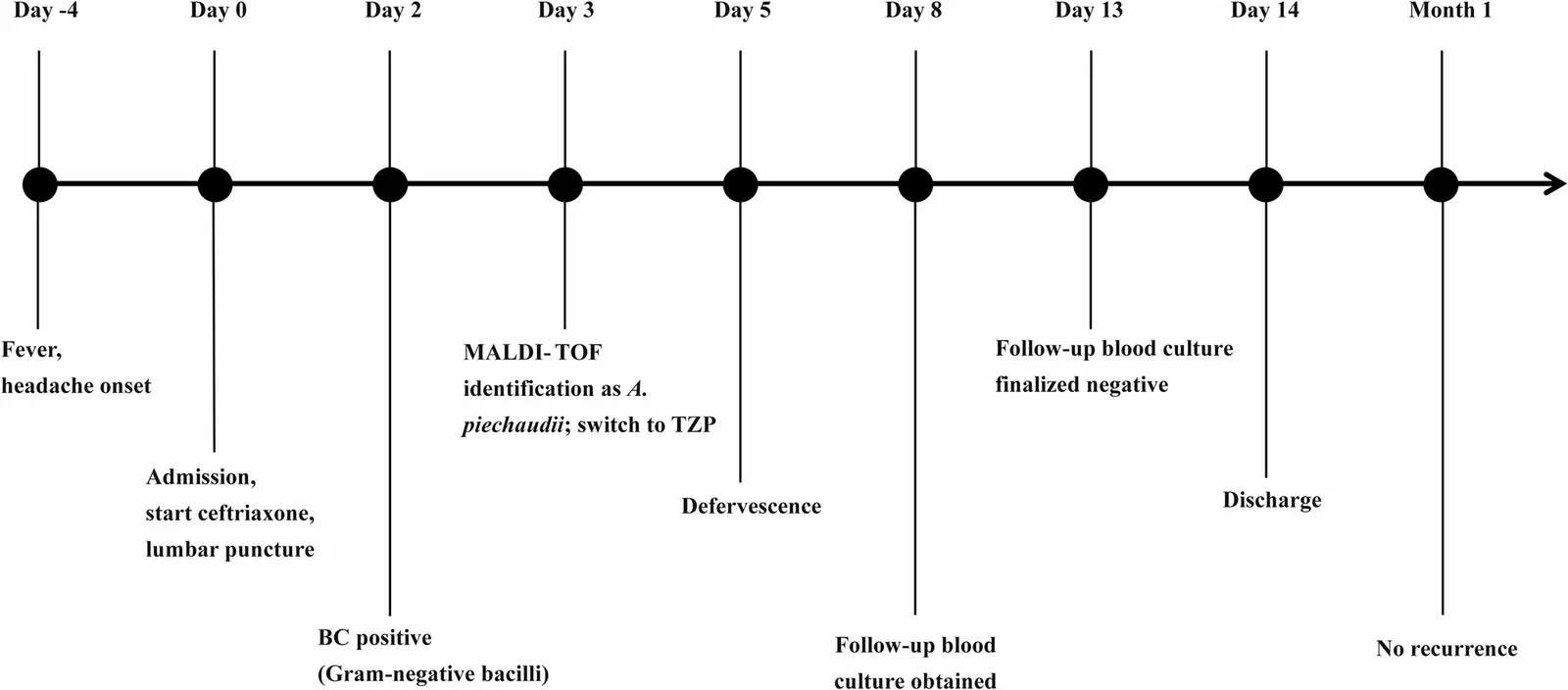
Clinical timeline of the case.Timeline displaying the key clinical events from symptom onset to follow-up. Abbreviations: BC, blood culture; MALDI-TOF MS, matrix-assisted laser desorption ionization time-of-flight mass spectrometry; TZP, piperacillin-tazobactam

On day 5 of hospitalization, the child’s fever resolved. Follow-up complete blood count showed white blood cell count 7.45 × 10⁹/L, neutrophil percentage 74.9%, C-reactive protein 9.44 mg/L, and procalcitonin 1.57 ng/mL. CSF culture showed no bacterial growth, ruling out intracranial infection. The nuchal rigidity observed at presentation resolved rapidly after admission and was suspected to be related to nonspecific meningeal irritation secondary to the systemic infection, although the precise mechanism could not be determined. Extended immunologic evaluation showed negative HIV antibody, normal complement C3 and C4 levels, and normal lymphocyte subsets and immunoglobulin levels. Neutrophil oxidative burst testing was not performed, so an occult phagocyte defect could not be completely excluded. Piperacillin-tazobactam monotherapy was continued. The child’s headache and vomiting completely resolved, and his mental status and appetite improved significantly. Follow-up blood inflammatory markers returned to normal, A follow-up blood culture obtained on hospital day 8 was negative and was finalized on hospital day 13. He was discharged on day 14 of hospitalization with full recovery. At one-month follow-up after discharge, the child was doing well with no recurrence or sequelae. A clinical timeline is provided in Fig. 1.

## Discussion

The genus *Achromobacter* comprises aerobic, non-fermenting, oxidase-positive Gram-negative bacilli that are widely distributed in soil and water environments. The taxonomy of this genus is complex and continues to evolve, now encompassing over 20 species, with *Achromobacter xylosoxidans* being the most common clinical isolate [3–6]. Common infection types include bloodstream infection, pneumonia, and catheter-related infection. A 2025 decade-long multicenter study showed that among a bloodstream infection cohort, 73.1% had a central venous catheter and 59.6% were immunocompromised [6]. A study by Gabrielaite et al. in 2021 indicated that colonization and infection of the genus *Achromobacter* in cystic fibrosis patients have been extensively studied, but epidemiological data in non-cystic fibrosis and immunocompetent populations are extremely scarce [7]. Although Achromobacter (formerly Alcaligenes) infections have been documented in children with various clinical presentations, including those without identifiable risk factors [8], pediatric cases caused by the rare species *A. piechaudii* have not been previously reported.

*Achromobacter piechaudii* is an extremely rare species, classified within the genus based on 16 S rRNA gene sequencing [9]. Only a few human cases were reported between 1998 and 2012 [1, 2, 10]. Our patient showed no evident immunodeficiency on routine evaluation, although an occult phagocytic defect could not be completely excluded because neutrophil oxidative burst testing was not performed. The child developed primary *Achromobacter piechaudii* bloodstream infection, representing the probable first pediatric case to our knowledge. This finding extends the host range of invasive disease caused by *Achromobacter piechaudii* from previously reported immunocompromised adults to apparently healthy children, suggesting that clinicians should include rare species of the genus *Achromobacter* in the differential diagnosis when managing pediatric bloodstream infection without obvious immunodeficiency [11].

Although a detailed history did not identify a clear source, we suspect that transient, unnoticed environmental exposure led to the infection.*Achromobacter* species are ubiquitous in water and soil; brief contact with contaminated surfaces followed by hand-to-mouth transmission or skin penetration could occur without the patient or family recalling a specific event. Because the child was febrile upon admission, hospital acquisition is unlikely. We therefore classify this as a community-acquired primary bloodstream infection.

Differentiating true bacteremia from contamination in children is challenging and requires correlation of clinical and laboratory findings [12]. In this case, both aerobic blood culture bottles turned positive within 14.2 h, the patient had high fever and elevated inflammatory markers, and defervescence occurred promptly after starting piperacillin-tazobactam without source control procedures. No other pathogens were recovered. These features strongly support a true infection rather than contamination.

Unlike previously reported *A. piechaudii* strains, which were largely susceptible [1, 2], our isolate had a multidrug-resistant phenotype. Based on current CLSI breakpoints for “Other Non-Enterobacterales”, it was susceptible only to piperacillin-tazobactam and resistant to several agents, including carbapenems and trimethoprim-sulfamethoxazole. This MDR classification should be considered provisional, as species-specific breakpoints are lacking. The resistance pattern also differs from the generally high susceptibility to carbapenems and trimethoprim-sulfamethoxazole seen in large *Achromobacter* series [6]. Cefiderocol may be a potential option for difficult-to-treat multidrug-resistant infections in children, as recent pediatric data support its activity against carbapenem-resistant Gram-negative bacteria [13].

This report has limitations. We did not perform molecular confirmation by *nrdA* sequencing [14, 15], whole-genome sequencing for resistance determinants, or neutrophil oxidative burst testing. These gaps mean that definitive species identification, full characterization of resistance mechanisms, and complete exclusion of an occult immunodeficiency could not be achieved.

## Conclusion

This case report highlights that multidrug-resistant *Achromobacter piechaudii* can cause primary bloodstream infection in apparently immunocompetent children. The case extends the clinical spectrum of this pathogen and underscores the importance of accurate species identification and susceptibility-guided therapy. Because species-specific breakpoints are lacking and molecular confirmation was not performed, the MDR phenotype should be interpreted cautiously. Further cases and genomic studies are needed to clarify the epidemiology and optimal management of *A. piechaudii* infections in children.

## Supplementary Information

Supplementary Material 1.

Supplementary Material 2.

## Data Availability

No datasets were generated or analysed during the current study.
