# Supplementary material for: Probable primary bloodstream infection caused by multidrug-resistant Achromobacter piechaudii in an apparently immunocompetent child: a case report
Source: BMC Pediatr. 2026 Aug 10;26:1134. doi: 10.1186/s12887-026-07498-8 (PMC13746578; doi:10.1186/s12887-026-07498-8)
Supplement: Supplementary file 2 [file 12887_2026_7498_MOESM2_ESM.pdf]

[18-May-2026]
Dear Editor,

We are submitting our manuscript entitled "**Primary multidrug-resistant Achromobacter piechaudii bloodstream infection in an immunocompetent child: A case report**" for consideration for publication in BMC Pediatrics.

Achromobacter piechaudii is an extremely rare opportunistic pathogen in humans, and all previously reported cases of invasive infection caused by this organism have been confined to adults with severe underlying diseases or immunocompromised conditions. Here, we report the first documented case worldwide of primary bloodstream infection caused by multidrug-resistant Achromobacter piechaudii in an immunocompetent child. The patient, a previously healthy 10-year-10-month-old boy with normal immunological function, presented with fever, headache, and signs of sepsis. Blood culture identified Achromobacter piechaudii, and antimicrobial susceptibility testing revealed a striking multidrug-resistant phenotype. Targeted therapy with piperacillin-tazobactam led to complete clinical recovery.

This case is of particular importance to the pediatric community for the following reasons: First, it challenges the traditional understanding that Achromobacter piechaudii only causes invasive disease in immunocompromised adults, expanding the host spectrum of this rare pathogen to healthy children. This finding has direct implications for the differential diagnosis of bloodstream infections in the pediatric population. Second, the multidrug-resistant phenotype of this isolate contrasts sharply with the generally susceptible patterns reported in the limited previous literature, providing an early warning signal regarding the evolving resistance profile of the genus Achromobacter. The clinical outcome also demonstrates the critical role of susceptibility-directed precision anti-infective therapy in pediatric rare bacterial infections.

We believe this manuscript is well suited for BMC Pediatrics, which has a dedicated scope in pediatric infection and welcomes studies that explore the epidemiology, pathology, prevention, and control of infectious diseases in neonates, children, and adolescents. Consistent with the journal‘s editorial philosophy, we present this case not on the basis of its perceived impact but because it is scientifically valid—with rigorous microbiological identification, verified antimicrobial susceptibility testing, and comprehensive immunological evaluation—and fills an important knowledge gap in the existing literature. The CARE guidelines for case reports have been followed, and the completed CARE checklist is included as a supplementary file.

We confirm that this manuscript has not been published elsewhere and is not under consideration by any other journal. All authors have read and approved the final version of the manuscript and agree with its submission to BMC Pediatrics. The authors declare no competing interests. Written informed consent for publication of

this case report has been obtained from the patient's parents, and a copy of the consent form is available upon request. No external funding was received for this study. We have also submitted a discretionary APC waiver application at the point of manuscript submission in accordance with the journal's open access policies.

We appreciate your time and consideration and look forward to your response.

Sincerely,

Yunfeng Xiang
Address: Department of pediatric Critical Care, Chongqing University Three Gorges Hospital, Chongqing, Wanzhou, China
Tel:13765273213/15330404448
Fax: 0085+023+58103050
Email: 1015464781@qq.com
